# [Expression of Concern] Glibenclamide exacerbates adriamycin-induced cardiotoxicity by activating oxidative stress-induced endoplasmic reticulum stress in rats

**DOI:** 10.3892/etm.2026.13092

**Published:** 2026-02-04

**Authors:** Meng-Lin Liu, Meng-Long Wang, Jing-Jun Lv, Jie Wei, Jun Wan

Exp Ther Med 15:3425–3431, 2018; DOI: 10.3892/etm.2018.5862

Following the publication of this paper, it was drawn to the Editor’s attention by a concerned reader that the control GAPDH western bands shown in Fig. 3A were strikingly similar to the control GAPDH western bands shown in Fig. 4A of a paper that had also been published in *Experimental and Therapeutic Medicine* by the same research group 2 years previously to this one [Liu ML, Wang ML, Liu JF, Luo Z, Shi L, Feng Y, Li L, Xu L and Wan J: Impact of ethyl pyruvate on Adriamycin-induced cardiomyopathy in rats. Exp Ther Med 12: 3201-3208, 2016]. Similarly, the control GAPDH western blots in Fig. 5B of the above paper were strikingly similar to the control GAPDH blots in Fig. 2C of the same previously published paper. Finally, upon performing an independent analysis of the data in this paper in the Editorial Office, it came to light that the AT6α bands in Fig. 3A were apparently the same as the protein bands that had been included in Fig. 2C of the previously published paper to portray the Nox2 western blot experiments.

Owing to the fact that the Editorial Office has been made aware of potential issues surrounding the scientific integrity of the above paper, we are issuing an Expression of Concern to notify readers of this potential problem while the Editorial Office continues to investigate this matter further.

